# Integrative Analysis of Transcriptomic and Methylomic Data in Photoperiod-Dependent Regulation of Cucumber Sex Expression

**DOI:** 10.1534/g3.118.200755

**Published:** 2018-10-30

**Authors:** Yun-Song Lai, Wei Zhang, Xiaohui Zhang, Di Shen, Haiping Wang, Yang Qiu, Jiangping Song, Xixiang Li

**Affiliations:** * Institute of Vegetables and Flowers, Chinese Academy of Agricultural Sciences, Beijing 100081, China; † Institute of Pomology & Olericulture, Sichuan Agricultural University, Chengdu 611180, China

**Keywords:** Cucumber, Photoperiod, Sex expression, DNA methylation, Transcriptome

## Abstract

The cucumber (*Cucumis sativus*) is characterized by its diversity and seasonal plasticity in sexual type. A long day length condition significantly decreased the cucumber female flower ratio by 17.7–52.9%, and the effect of photoperiod treatment is more significant under low temperature than under high temperature. Transcriptome analysis indicates that the photoperiod treatment preferentially significantly influenced flower development processes, particularly MADS-box genes in shoot apices. The long-day treatment resulted in predominantly transposable element (TE)- and gene-associated CHH-types of DNA methylation changes. Nevertheless, there was significant enrichment of CG- and CHG-types of DNA methylation changes nearing transcription start sites (TSSs)/transcription end sites (TESs) and gene bodies, respectively. Predominantly negative association between differentially methylated regions (DMRs) and differentially expressed genes (DEGs) were observed which implied epiregulation of DEGs. Two MADS-box genes that were significantly downregulated by long photoperiod showed significant hypermethylation in promoter regions that is essentially TE-rich. This study indicates MADS-box genes which are partially regulated by promoter methylation state may mediate photoperiod-dependent regulation of cucumber sex expression.

Photoperiodic cues from light/dark cycles help plants to predict environmental change and thereby regulate their developmental processes under favorable/unfavorable conditions. Plants sense the light environment via the leaves and then measure changes in day length using circadian clocks (Song *et al.* 2010; Kinmonth-Schultz *et al.* 2013). The major physiological responses to photoperiod condition changes include alterations in flowering time, hypocotyl elongation, and reactive oxygen species (ROS) homeostasis (Shim and Imaizumi 2015). In addition, plant sex determination is affected by the photoperiod condition, which is a known type of environmental sex determination (ESD). Essentially, short days promote femaleness in many plants that have unisexual flowers, *e.g.*, cucumbers (*Cucumis sativus*) (Cantliffe 1981), melon (*Cucumis melo*) (Rudich and Peles 1976), and *Atriplex halimus* (Talamali *et al.* 2002). Accordingly, most cucumber germplasm accessions show significant, drastic decreases in female flowers in early autumn (Dou *et al.* 2015), and the long day length condition in early autumn might be the major reason.

Cucumber is a model plant in the field of plant sex expression whose regulation mechanism is now quite clear. Ethylene is the “sex hormone”, and four “sex genes” have been identified: *F/CsACS1* (Trebitsh *et al.* 1997; Mibus and Tatlioglu 2004; Knopf and Trebitsh 2006), *M/CsACS2* (Saito *et al.* 2007; Boualem *et al.* 2009; Li *et al.* 2009), *A/CsACS11* (Boualem *et al.* 2015), and *G/CsWIP1* (Boualem *et al.* 2015; Chen *et al.* 2016). Recently, CsACO2 was confirmed to be critical in sex determination and catalyzes the last step of ethylene biosynthesis (Chen *et al.* 2016). In addition to known “sex genes”, *ethylene response 1* (*ERT1*), *ethylene sensitive 3* (*EIN3*), and *ethylene responsive factor 110* (*ERF110*) were reported to regulate cucumber sex expression (Wang *et al.* 2010; Tao *et al.* 2018; Liu *et al.* 2008). Yin and Quinn (1995) proposed a “one-hormone hypothesis” to highlight the dominant role of ethylene in cucumber sex expression (Yin and Quinn 1995). Nevertheless, gibberellins (GAs) that regulate flower development play a great role in sex expression that can be ethylene independent (Pimenta Lange and Lange 2016; Zhang *et al.* 2017). Based on the above progress, cucumber can be a model species in the study of plant ESD phenomena. By comparing the diurnal rhythm of ethylene accumulation and transcription level, *CsACS2* expression in shoot apices was suggested to mediate photoperiod-dependent ESD (Yamasaki *et al.* 2003).

Currently, an increasing number of studies are reporting that DNA methylation is involved in genetic sex determination (GSD) processes. Comparisons between the methylomes of male and female flowers indicate that a differential DNA methylation state at miRNA172, which targets *APETALA2* (*AP2*), may account for poplar GSD (Song *et al.* 2012b; Song *et al.* 2015). Hypomethylation of the *MeG*I promoter results in female flower formation in persimmon (Akagi *et al.* 2016). The evolution of sex chromosomes in dioecious plants, such as *Carica papaya*, *Silene latifolia* and *Rumex acetosa*, is enforced by heterochromatinization resulting from DNA methylation (Zhang *et al.* 2008; Li *et al.* 2016b). In melon (*Cucumis melon*), a close relative to cucumber, DNA hypermethylation of the *CmWIP1* promoter, due to a transposable element (TE) insertion, leads to the transition of flowers from male to female (Martin *et al.* 2009). These above studies stress the important role of DNA methylation-based epiregulation. Histone modification participates in flower development as well as sex determination (Guo *et al.* 2015; Latrasse *et al.* 2017), indicating a coordinated regulation of DNA methylation and histone modification.

Since sex expression can be genetically controlled by DNA methylation state-related epigenetic sites, it is highly likely that DNA methylation plays an important role in plant ESD. The reason is that plant genome-wide DNA methylation is known to display a high level of plasticity in response to environmental stimuli, such as NaCl stress (Baek *et al.* 2011; Song *et al.* 2012a; Jiang *et al.* 2014), drought stress (Wang *et al.* 2016; Chwialkowska *et al.* 2016), phosphate starvation (Yong-Villalobos *et al.* 2015), the pesticide atrazine (Lu *et al.* 2016), heat stress (Popova *et al.* 2013), nematode infection (Rambani *et al.* 2015) and bacterial pathogen exposure (Dowen *et al.* 2012; Yu *et al.* 2013), although the mechanism of the interaction between stresses and epigenetic control is far to be known (Annacondia *et al.* 2018). In fact, plants benefit from long-term environmental adaption provided by spontaneous epimutation, which can contribute to epigenetic and genetic variance or even new gene formation (Sahu *et al.* 2013; Silveira *et al.* 2013), although many epimutations can be lost in subsequent generations (Jiang *et al.* 2014). The *ACO* gene and *APETALA2/ethylene response factor* (*AP2/ERF)* gene might be epicontrolled and may account for temperature-dependent regulation of sex expression in cucumbers (Lai *et al.* 2017).

In the present study, we profiled the responsive patterns of the methylome and transcriptome in a photoperiod treatment, which provided information about the regulation mechanism of the photoperiod-dependent regulation of sex expression in cucumbers.

## Materials And Methods

### Plant materials

Chinese Long cucumber, also named “9930”, was used to survey the effect of photoperiod treatment on the methylome and transcriptome. High-temperature (32°/24°, day/night) and long-day (16 h/8 h, day/night) (HL) treatment was performed from the beginning of sowing to couple with previously published high-temperature and short-day (8 h/16 h, day/night) (HS) treatment. Low-temperature (23°/15°, day/night) and long-day (LL) treatment was performed to couple with previously published low-temperature and short-day (LS) treatment (Lai *et al.* 2017). This is to evaluate the photoperiod effect under either high temperature or low temperature. A high temperature is unfavorable for female flower formation (Lai *et al.* 2017).

### Sampling and investigation method

When the fourth true leaves had unfolded, shoot apices of the treated “9930” plants that had not developed any visible flowers were carefully dissected under a microscope and immediately frozen in liquid nitrogen. The sampling for each treatment was performed in triplicate. In total, more than 1000 young plants were used for the sampling of shoot apices for each replicate. Pooled samples of 500 shoot apices per replicate were used for total RNA isolation, followed by mRNA-seq. The other 500 shoot apices from each replicate were pooled as a single sample and used for DNA isolation followed by whole-genome bisulfite (BS) sequencing (WGBS). Meanwhile, the other seedlings were transplanted into the same condition (temperature, 20-35°; photoperiod, 12h/12h) in a greenhouse to investigate femaleness.

### Whole-genome bisulfite sequencing (WGBS) and data processing

Deep sequencing and read mapping and processing were performed at BGI-Shenzhen (Shenzhen, China). The methods for DNA extraction, library construction, and sequencing data processing were the same as described previously (Lai *et al.* 2017). Briefly, 5 µg of genomic DNA was subjected to BS treatment using the EpiTect Bisulfite Kit, which converts any unmethylated cytosines to thymidines. The BS conversion rates in the WGBS of the HS and HL treatments were both 99.51%. To evaluate the conversion efficiency, 25 ng of phage DNA was used as an internal reference. The WGBS DNA library templates obtained were quantified via electrophoresis of an aliquot of the templates on an Agilent Technologies 2100 Bioanalyzer using a High-Sensitivity DNA chip. Sequencing was performed using an Illumina Genome Analyzer IIx according to the manufacturer’s instructions.

Before alignment to the genome, all Cs in the forward clean reads were altered to Ts, and all Gs in the reverse clean reads were altered to As to maintain consistency with the BS conversion. The clean reads were then aligned with the Chinese Long *Cucumis sativus* genome (version 2.0; http://cmb.bnu.edu.cn/Cucumis_sativus_v20/) using BSMAP (Xi and Li 2009). Only reads that were unique matches and only cytosines that were covered by at least four reads were processed in the subsequent steps.

Methylcytosines (mCs) were identified as described previously (Lister *et al.* 2009). The number of methylation-supporting reads of an mC was required to be at least the anticipated number in a binomial test adjusted by the BS conversion rate. Identification of differentially methylated cytosine (DmCs) between two treatments was performed using *Fisher’s exact test*. Cytosine sites with a *p*-value < 0.05 and changes in methylation levels of at least 20% were identified as DmCs. Differentially methylated regions (DMRs) were identified as previously described, with modifications (Gao *et al.* 2014). For symmetric CG and CHG sequences, DMRs were screened across the Watson strand, and the DNA methylation of cytosines on the Crick strand was not interrogated; for asymmetric CHH sequences, DMRs were separately screened across the Watson and Crick strands. First, five adjacent CG/CHG/CHH motifs containing at least four CG/CHG/CHH sequences with the same response pattern and Wilcoxon rank-sum test *p*-values < 0.05 were considered candidate DMRs. Next, 3′ downstream adjacent CG/CHG/CHH sequences with the same response patterns were incorporated with the candidate DMR until differential significance disappeared. The distance between two adjacent CG/CHG/CHH sequences should be less than 200bp in the DMR identification process. DMRs smaller than 50bp in length and for which the methylation level differences were smaller than 0.1 were discarded.

### Transcriptome sequencing and data processing

The methods for RNA extraction, library construction, and sequencing data processing were as described previously (Lai *et al.* 2017). Briefly, 1 µg of total RNA was used to enrich poly(A) mRNAs, which were then fragmented and ligated to an adaptor. Sequencing was performed using an Illumina Genome Analyzer IIx according to the manufacturer’s instructions. Clean reads were mapped to the Chinese Long *Cucumis sativus* genome version 2.0 (version 2.0; http://cmb.bnu.edu.cn/Cucumis_sativus_v20/) using BWA (Li and Durbin 2009). The transcripts were annotated after assembling by referring to the annotation file “Cucumber_v2.gff3” (version 2.0; http://cmb.bnu.edu.cn/Cucumis_sativus_v20/). The transcript levels were calculated as fragments per kilobase of transcript per million fragments (FPKM) using the Cufflinks software package (Mortazavi *et al.* 2008). Differentially expressed genes (DEGs) were identified using the Noiseq package, and the criteria were a divergence probability ≥ 0.8 and a fold-change ≥ 2 (Audic and Claverie 1997; Benjamini *et al.* 2001). We normalized TE transcripts to the total number of reads aligned for each TE and expressed this value as FPKM (Jin *et al.* 2015). Adjusted *p*-values (<0.05) were used to determine the statistical significance of differentially expressed TEs (DETs). Transposons that overlapped with protein-coding genes were discarded.

### Associations between DmCs/DMRs, genes, and TEs

The position of TEs and gene structures was determined by referring to the cucumber genome annotation (version 2.0; http://cmb.bnu.edu.cn/Cucumis_sativus_v20/). TSS and TES are simply determined as the boundary of an mRNA in the annotation. A C/mC/DmC is allowed to be collated in more than one genomic feature, *e.g.*, TEs, noRNA, genic regions, intergenic regions, CDS, introns, UTRs, TEs, 2-kb upstream regions, and 2-kb downstream regions (Figure 2A). A genic region includes the gene body and the surrounding 2-kb regions. A TE includes only the body. The regions that are more than 5 kb from the transcription start site (TSS) or transcription end site (TES) were defined as intergenic regions. To stress the detailed association with cytosine methylation change, a gene body was subdivided into 5 equally sized bins, which together with 10 bins (bin = 300 bp) upstream or downstream of a gene were used for enrichment analyses. In the enrichment analyses, DmC fold enrichment value = (DmC_feature_ / DmC_total_) / (mC_feature_ / mC_total_). The enrichment significance is indicated by *p*-values derived from the hypergenometric test. The position of a DMR relative to gene structures was determined relative to the midpoint of each DMR (Figure 2B). DMRs were first defined as genic DMRs or intergenic DMRs based on the position of the DMRs relative to genes. Each genic DMR was then only assigned to the nearest genes. The association of a DMR with a TE was confirmed if the regions overlapped (Figure 2C).

### Data availability

The WGBS data were deposited in the NCBI SRA (https://www.ncbi.nlm.nih.gov/sra/) under accession numbers SRR5431155 (HL) and SRR5430207 (LL) in this study. The WGBS sequencing data of HS and LS were retrieved from NCBI SRA with the accession IDs SRR5430777 (HS) and SRR5430103 (LS). The transcriptome sequencing data of HL and LL were deposited in the NCBI SRA in this study. The accession IDs of HL replicates are SRR6837906, SRR6837907, and SRR6837908; those of LL replicates are SRR6837824, SRR6837841, and SRR6837842. The transcriptome sequencing data of HS and LS were retrieved from NCBI SRA. The accession IDs of three HS replicates are SRR5462513, SRR5462516, and SRR5462554; those of three LS replicates are SRR5460753, SRR5461296, and SRR5461309. All data are publicly available. Figure S1 shows the ranges of methylation change extent for the three sequence contexts. Figure S2 shows gene classification of DEGs resulting from the long-day treatment based on GO terms. Table S1 and S2 show details of the MethylC-Seq libraries and methylation rates of different genomic structures. Table S3 shows the DEGs in the photoperiod treatment.Supplemental material available at Figshare: https://doi.org/10.25387/g3.7207718.

## Results

### Long day length treatment significantly decreases female flower ratio

In nature, temperature and photoperiod are the most important factors in a seasonal shift. In this study, the effect of photoperiod on cucumber femaleness was confirmed in incubators, in addition to the previously reported temperature treatment (Lai *et al.* 2017). As a result, long-day treatments resulted in significant decreases of 52.9% (*P* < 0.01) and 17.7% (*P* < 0.5) in the proportion of nodes with pistillate flowers (PNPF) value under low temperature and high temperature conditions, respectively.

### A long photoperiod induces a CHH-type methylation change

**Figure 1:**
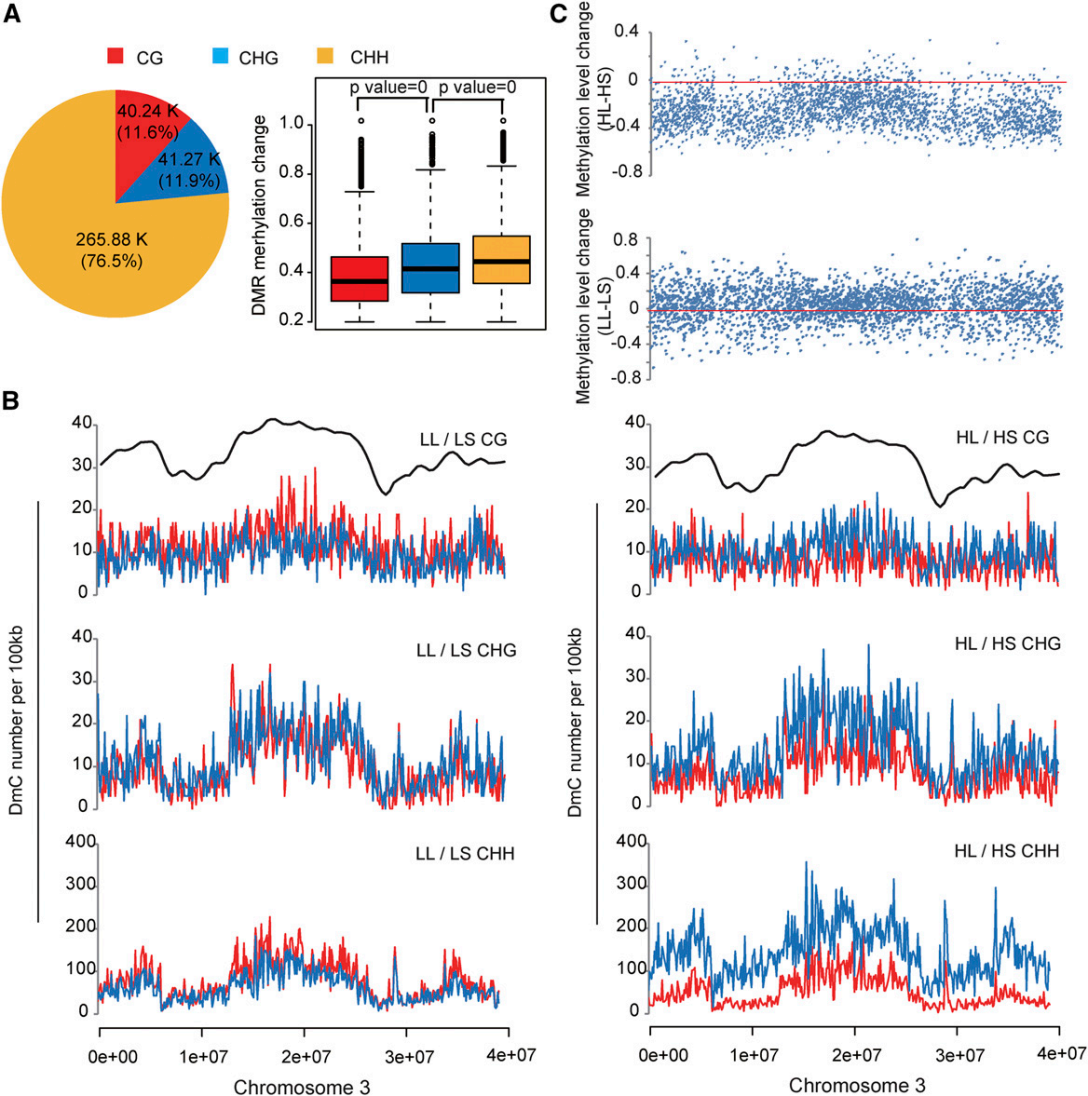
Long-day-induced differential methylation. (A) Number and proportion of CG-, CHG-, and CHH-types of DmCs in long-day-length treatment under low temperature (left). K, thousand. Extent of change in the methylation level in three sequence contexts (right). Box plot: blank boxes, 25–75% quartiles; blank lines within blank boxes, median of distribution (50% quartile). *p*-values were derived from a *t*-test between sequence contexts. (B) Distribution of number of DmCs on chromosome 3. Red lines, hypermethylated DmCs; blue lines, demethylated DmCs. The y-axes show the DmC number in a bin of 10 kb. Dark lines only show the profile of the mC/C ratio, bin = 100kb. (C) Distribution of the extent of methylation changes in CHH-type DmCs on chromosome 3. The methylation level values of HL and LL were subtracted by those of HS and LS, respectively. Bin = 10 kb. LL, low temperature and long day length; LS, low temperature and short day length; HL, high temperature and long day length; HS, high temperature and short day length.

Shoot apices of ‘9930’ plants that did not develop any visible flowers during the photoperiod treatments were carefully dissected under a microscope and subjected to DNA extraction. The morphological divergence of male and female flowers begins at stage 6, and sex reversal does not occur after this stage (Bai and Xu 2013). The sequencing data information from the whole-genome bisulfite sequencing (WGBS) is shown in Table S1. We compared the DNA methylation landscape between short (8 h) and long (16 h) photoperiod conditions at the single-nucleotide level and identified differentially methylated cytosines (DmCs). The photoperiod cue resulted in major responses at the CHH sites in terms of both the abundance and the extent of methylation change (Figure 1A, Figure S1 and Table S2). Differentially methylated regions (DMRs) determined using a sliding-window approach showed a consistent dynamic DNA methylation change with DmCs (Figure 1A).

We further profiled methylation changes across the chromosomes. Taking the longest chromosome, chromosome 3, as an example, the putative mC-rich central chromosome region showed much a higher DmC density than the arms, especially for CHG- and CHH-DmCs (Figure 1B). Moreover, the methylation changes at CG-, CHG-, and CHH-DmCs were generally coordinated with each other across chromosomes; hypermethylation and hypomethylation were also coordinated with each other. Intriguingly, the extent of methylation change at CHH among the treatments was chromosome feature related. In the comparison of high temperature and long day (HL) / high temperature and short day (HS), where HS was taken as a baseline, there was a genome-wide reduction in methylation, but the chromosome arms that were less methylated showed a larger reduction in the methylation level than the central region of the chromosome (Figure 1C). In comparing low temperature and long day (LL) / low temperature and short day (LS), the chromosome arms showed greater flexibility in methylation level change than the central region (Figure 1C). Because the chromosome arms were often gene-rich euchromatin regions, it is likely that the long-photoperiod-induced DNA methylation change was gene related.

### DNA methylation landscape reshaping is closely associated with genes and TEs

**Figure 2:**
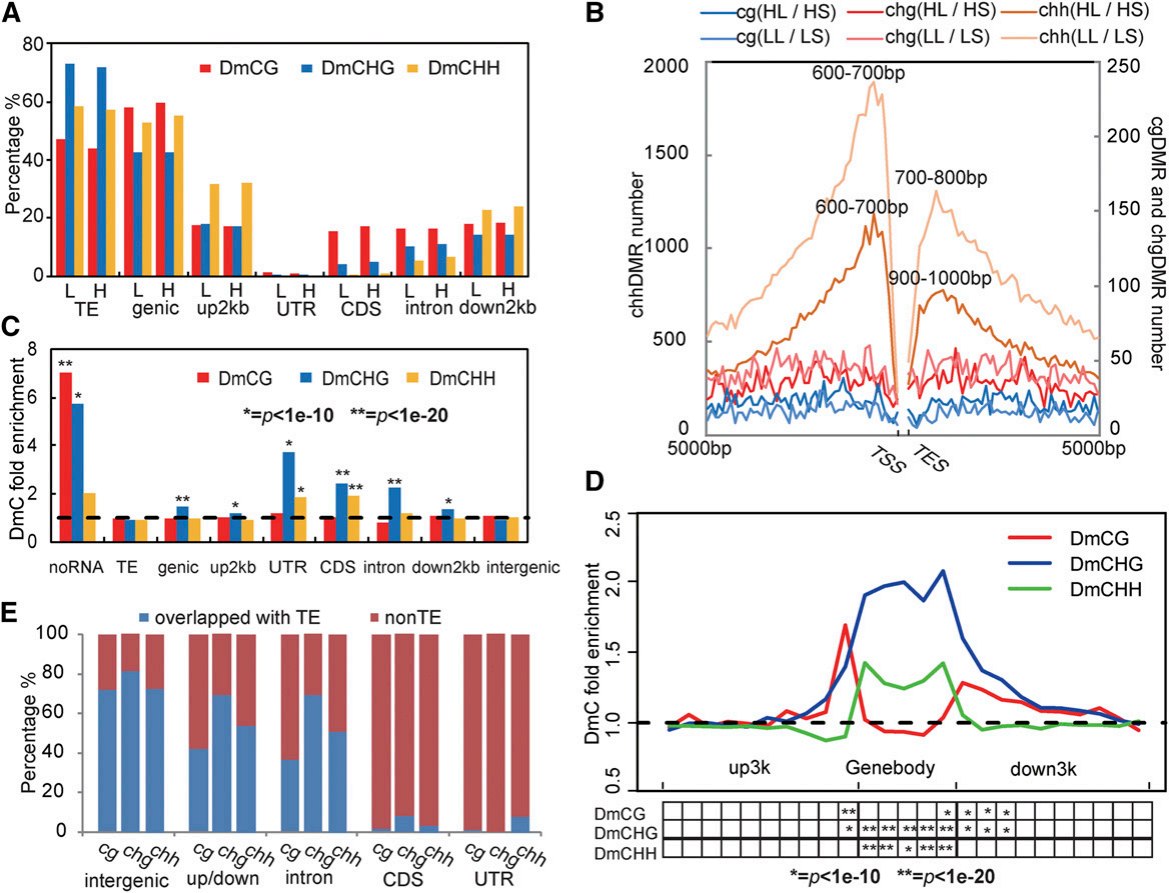
Association of photoperiod stimuli-induced DMRs with genes and TEs. (A) Proportion of photoperiod condition-induced DMRs in different genomic features. TE, transposable elements; gene, protein-coding genes; UTR, untranslated regions; CDS, coding sequence; up5kb, regions 5-kb upstream of a transcription start site (TSS); down5kb, regions 5-kb downstream of a transcription end site (TES). L, long-day-length treatment under low temperature condition; H, long-day-length treatment under high temperature condition. (B) Enrichment of DMRs with increasing distance from gene TSSs/TESs. Bin = 100 bp. (C-D) Enrichment of DmCs (induced by long photoperiod under low temperature) at indicated genomic features (C) and in gene bodies, as well as upstream and downstream regions (D). Each gene body was subdivided into 5 equally sized bins; for upstream and downstream regions, bin = 300 bp. *p*-values were derived from a hypergenometric test between the numbers of DmCs and mCs with each genomic feature, genic feature or bin. (E) Percentage of DMRs that overlapped with any TE.

We further inspected the distribution patterns of differential methylation in the genome resulting from the long-day treatment. Approximately half of the DMRs resulting from the photoperiod treatment occurred in genes or their nearby regions (Figure 2A). CG-DMRs notably occurred in CDS and intron regions and in nearby gene regions. CHH-DMRs were more associated with nearby gene regions than were CG- and CHG-DMRs. Specifically, CHH-DMR abundance was related to the distances from transcription start sites (TSSs)/transcription end sites (TESs) (Figure 2B). The abundance of CHH-DMRs increased rapidly with the distance from a TSS/TES up to 100 bp, and there was a particular enrichment in the range of 600-1000 bp from TSSs/TESs. CG- and CHG-DMRs did not clearly show this trend. Consistent with the above results, we observed strong enrichment of DmCs in genic features, including the untranslated region (UTR), CDS, intron, and surrounding 2-kb regions (Figure 2C). In detail, there is a significant enrichment of DmCHG in the gene body and the adjacent 300-600 bp; regarding DmCHHs, they are significantly enriched in gene bodies. There is significant enrichment of DmCGs at the boundary of gene bodies, namely, UTRs, but not in any other regions (Figure 2D).

The high proportions of TE-DMRs in all three of these sequence contexts indicate that TEs play a central role in DNA methylation differences (Figure 2A). Notably, over 72% of the CHG-DMRs overlapped with TEs. Moreover, a high proportion of DMRs in intergenic regions, nearby gene regions and introns overlapped with TEs, in sharp contrast with those in CDS and UTRs, indicating that distinct mechanisms might reshape these regions (Figure 2E). To further analyze the association of DMRs and TEs, we screened differentially expressed TEs (DETs) using RNA-seq data. A total of 10 DETs, 9 upregulated LTR-type TEs and 1 downregulated unknown TE were identified between the LS and LL conditions (Table 1). In contrast, only 1 DET was identified between the HS and HL conditions, and it was upregulated. All DETs were located in four regions: two euchromatin regions on chromosome 2, one heterochromatin region on chromosome 4, and one heterochromatin region on chromosome 6. Three uncharacterized genes (Csa4M243140.1, Csa6M239685.1, and Csa6M239680.1) were upregulated near the DETs. Notably, all the DETs were associated with DMRs, among which one DET (Chr4.11418) was associated with as many as 12 DMRs, strongly supporting a relationship between DETs and DMRs.

**Table 1:** Associations of DMRs and DETs

TE ID	TE type	LS^1^	LL^1^	DMR position	type
Chr2.1737	unknown	21.06	2.29	2895044-2895139	CG/down
Chr2.18932	LTR/Gypsy	23.51	151.09	19247391-19247471	CHH/down
				19247523-19247585	CHG/up
				19247572-19247676	CG/up
Chr4.11395	LTR/Gypsy	1344.08	7392.52	10157041-10157162	CHH/up
				10157974-10158139	CG/down
				10158526-10158581	CG/up
				10159108-10159175	CHH/down
				10160828-10160883	CG/up
				10161255-10161388	CG/down
				10166764-10167158	CG/up
Chr4.11405	LTR/Gypsy	337.88	1704.24	10164610-10164937	CG/up
Chr4.11411	LTR/Gypsy	0.32	38.66	10166764-10167158	CG/up
Chr4.11414	LTR/Gypsy	2.57	103.78	10170488-10170582	CG/up
				10170660-10170721	CG/up
				10171222-10171380	CG/up
				10171278-10171358	CHG/up
Chr4.11418	LTR/Gypsy	1581.50	8599.17	10174326-10174440	CHG/up
				10175334-10175441	CG/up
				10176583-10176641	CG/up
				10178622-10178718	CG/down
				10178873-10178967	CG/down
				10179267-10179323	CHG/down
				10179845-10179931	CG/down
				10180277-10180350	CHG/up
				10180434-10180598	CG/up
				10180810-10180884	CHG/down
				10181086-10181622	CG/down
				10181372-10181582	CHH/up
Chr4.11442	LTR/Gypsy	41.50	293.74	10189425-10189483	CG/up
Chr6.14802	LTR/Gypsy	1268.47	7504.71	13602334-13602466	CG/up
				13602478-13602580	CG/down
Chr6.14804	LTR/Gypsy	837.91	5035.12	13603143-13603194	CG/down
				13603385-13604182	CG/down

1 Expression level of TEs is denoted in FPKM.

### Transcriptome change underlies photoperiod-dependent regulation of sex expression

The same shoot apices that were used for the WGBS were also used for mRNA-seq. To determine photoperiod-related changes in the transcriptome, we thoroughly compared HL with HS (HL/HS) and LL with LS (LL/LS) at the transcriptomic level. A total of 20,734 transcripts were detected in the cucumber shoot apices under long-day conditions. There were 138 significantly differentially expressed genes (DEGs) induced by the long photoperiod, and 109 genes were annotated using an nr Blast search (Table S3). Eleven DEGs were identified regardless of the temperature condition, and all of these exhibited the same up- or downregulation responses to the photoperiod treatments under both high temperature and low temperature, suggesting that these DEGs were strongly regulated by photoperiod cues.

**Figure 3:**
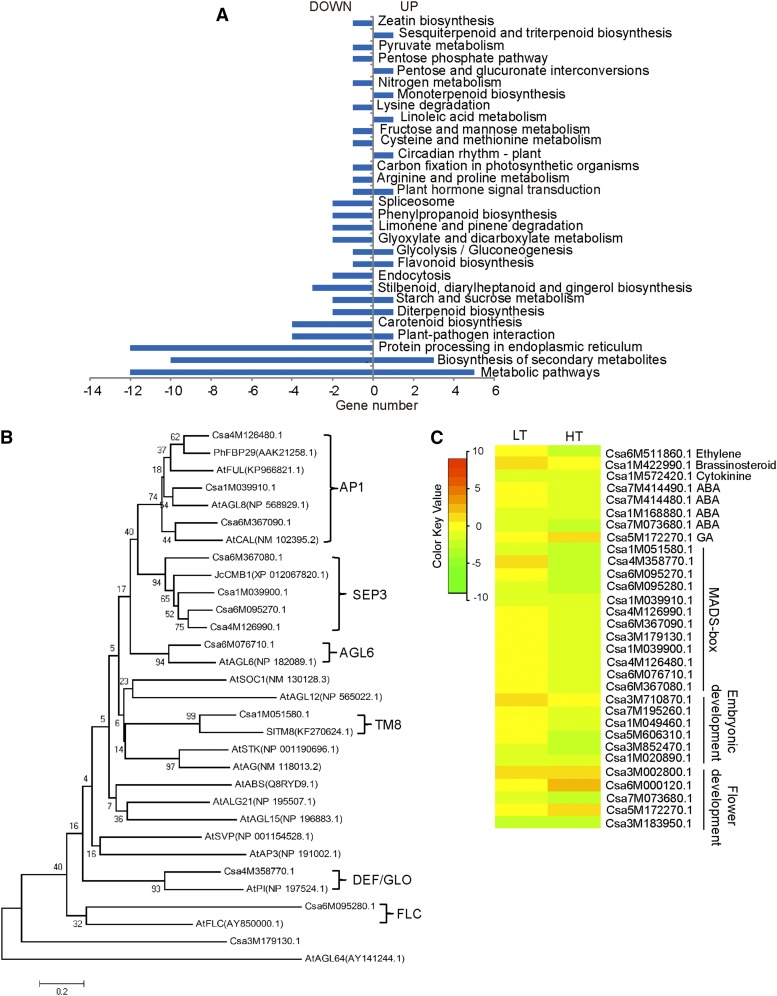
Transcriptomic changes in response to a change in the photoperiod condition. (A) Gene classification of DEGs resulting from long-day treatment based on KEGG pathway terms. (B) Neighbor-joining phylogenetic tree of differentially expressed MADS-box genes and their counterparts in Arabidopsis. Deduced amino acid sequences were retrieved from the TAIR database (https://www.arabidopsis.org/), and the accession ID numbers are shown. The bar indicates a genetic distance of 0.2. (C) Heatmap presentation of transcriptional changes in DEGs related to cucumber sex expression in the photoperiod treatment. LT, long-day-length treatment under low temperature, HT, long-day-length treatment under high temperature.

Notably, 20 of these 109 genes were assigned to “transcription factor activity,” and 24 genes were assigned to embryonic development, post-embryonic development or flower development according to a Gene Ontology (GO) analysis (Figure S2). Kyoto Encyclopedia of Genes and Genomes (KEGG) pathway analysis identified 44 genes involved in 30 pathway items (Figure 3A). There was a strong enrichment of genes that participate in metabolic pathways and protein processing in the endoplasmic reticulum, most of which were downregulated. A total of 12 MADS-box genes showed significant differential expression in response to the long-day treatment, which is a large number considering that there were only a total of 138 DEGs. Moreover, phylogenetic tree analysis indicated that 11 of these MADS-box genes were clustered with six known MIKC^c^ clade subgroups (Figure 3B). These MADS-box genes play important roles in flower development, some of which are putative class A, B, and E genes in cucumbers. Importantly, all of them were downregulated in the long-day-length treatment.

We further profiled the transcription level changes of DEGs in terms of MADS-box genes and other genes closely related to cucumber flower development and sex expression (Figure 3C). There were 31 DEGs resulting from photoperiod treatment that might be involved in photoperiod-dependent regulation of cucumber sex expression. It is likely that long-day treatment downregulated ABA signal transduction-related genes regardless of the temperature condition. Likewise, long-day treatment had an influence on flower development-related genes. The effect of photoperiod on the transcriptome is temperature dependent in most cases. Under high temperature, long-day treatment downregulated CsACO_2_ (Csa6M511860) and upregulated gibberellin 20 oxidase (Csa5M172270); under low temperature, long-day treatment upregulated brassinosteroid biosynthetic gene Csa1M422990. Notably, long-day treatment downregulated embryonic development-related genes and MADS-box genes under high temperature, while the downregulation trend of these genes was subtle under low temperature. These results indicate that phytohormone- and flower organ development-related genes were influenced by long-day treatment, and these transcriptome changes may account for the cucumber sex expression change. Importantly, the response pattern of these genes to photoperiod cues is dependent on temperature.

### Association of DNA methylation changes and transcriptome changes

Cytosine methylation state can determine gene activity, which is known as a kind of epigenetic regulation (epiregulation). CG- and CHG-type DMRs in CDS and nearby gene regions (<2 kb to TSS or TES) are negatively associated with transcription level changes in cucumbers (Lai *et al.* 2017). These types of negatively coordinated change may suggest epiregulation sites. There were a total of 28 interaction sites between DMR and DEG, and 75.0% (21 interaction sites) of them showed a negative association. A total of 17 DEGs showed the negative association of transcription level and methylation level. A total of 10 putative epicontrolled genes were annotated by nr Blast (Table 2). Notably, 2 MADS-box genes were downregulated by the long-day length, with a methylation level increase in nearby gene regions. The putative epiregulation of a *FLOWERING LOCUS C* (*FLC*) -like MADS-box protein gene (Csa6M095280.1) likely plays a role in photoperiod-dependent regulation of sex expression.

**Table 2:** Prediction of gene expression epiregulation in response to changes in environmental conditions

Gene ID	Gene annotation	Condition^1^ change	E-change^2^	DMR change^3^
Csa6M010000.1	acetyltransferase	LL/LS	−2.04	DOWN,CHG(1438;0.12)
Csa4M003670.1	Calvin cycle protein CP12-2	HL/HS	−1.82	CDS,CG(0.26)
Csa3M179130.1	MADS-box TF	HL/HS	−1.14	UP,CG(876;0.12)
Csa6M095280.1	MADS-box TF	HL/HS	−2.15	UP,CHG(1283;0.15)
Csa1M532310.1	MAPKKK	HL/HS	−2.72	UP,CG(1819;0.10)
Csa3M171860.1	MLP	HL/HS	1.21	UP,CG(1635;-0.11)
Csa5M606310.1	NAC domain protein	HL/HS	−1.84	CDS,CG(0.18)
Csa4M501830.1	phloem	HL/HS	−1.77	UP,CHG(1919;0.20)
Csa1M168880.1	secoisolariciresinol dehydrogenase	HL/HS	−1.17	CDS,CG(0.18)
Csa7M414480.1	short-chain dehydrogenase reductase 3b	HL/HS	−1.05	CDS,CG(0.14),CG(0.25)

1 LS or HS was taken as the baseline or control.

2 Expression change is denoted by the value of log_2_(Long photoperiod-FPKM/Short photoperiod-FPKM).

3 UP, upstream-positioned DMRs; DOWN, downstream-positioned DMRs; distance of upstream- and downstream-positioned DMRs relative to TSS/TES and change in the methylation level are shown in parentheses.

## Discussion

The effects of temperature and photoperiod on sex expression appear to be a common feature in plants. Short-day treatment promotes female and suppresses male sex expression in short-day and some day-neutral plants; low temperature promotes female sex expression, particularly when it occurs in the dark period of a daily photoperiodic cycle (Heslop-Harrison 1956). Most cucumbers are day-neutral plants, except for some cucumber groups, *e.g.*, Xishuangbanna, a semi-wide group that belongs to short-day plants. The long day condition has the same influence on female flower formation under high temperatures in cucumbers. However, the long-day treatment resulted in a decrease of as much as 52.9% in the PNPF values of “9930,” smaller than that observed with the high-temperature treatment (Lai *et al.* 2017). Indeed, the change in photoperiod conditions in nature is slow and gradual and, therefore, is less of a determinant than temperature in terms of plant survival. These findings are consistent with the more drastic changes in the methylome and transcriptome responses to temperature stimuli compared with those to photoperiod stimuli. The number of DMR-DEG associations is smaller in photoperiod treatment than in temperature treatment.

Here, we profiled and characterized the changes in transcriptome response to photoperiod condition change. This *in silico* analysis underlies a possible molecular mechanism of the cucumber sex expression change. Impressively, the long-day treatment significantly suppressed multiple flower development-related processes and particularly MADS-box genes. Environmental regulation of so many MADS-box genes may result in sex expression change via two mechanisms. One of the mechanisms is based on the regulation of ethylene signal pathway by MADS-box gene. *AP3* homologs in cucumbers that is a B-class MADS-box gene in ABC model negatively regulates *ETR1*, a negative regulator in ethylene signal pathway (Sun *et al.* 2016). This regulation is cucumber-specific and stamen-specific. Correspondingly, downregulation of MADS-box genes in long photoperiod condition probably decrease ethylene signal cascade which is necessary for stamen arrest and ovary development. For the other mechanism, flower bud differentiation is influenced in the photoperiod treatment first and that subsequent sex expression is thereafter affected. This tendency is particularly exemplified in the Xishuangbanna cucumber, which developed no flowers at all regardless of flower sex under the long-day treatment (data not shown). For “9930”, however, unknown mechanism must prevent male flower from photoperiod influence because the formation of male flowers are not affected. Basically, photoperiod cue may exert influence on cucumber sex expression via MADS-box genes, which is different from temperature cues. The temperature regulation of the flower architecture appears to involve multiple hormone signaling networks (Patel and Franklin 2009). In our previous study, temperature treatments likely affect cucumber sex expression mainly via ethylene biosynthetic and signal transduction pathways, although two MADS-box genes were affected at the transcription level (Lai *et al.* 2017).

One apparent common feature is that environmental factors mainly result in gene- and TE-associated DNA methylation changes, as illustrated in treatments with short-term heating(Li *et al.* 2016a), chilling (Zhang *et al.* 2016), herbicide application (Kim *et al.* 2017), and water deficit (Xu *et al.* 2018). In tomato fruits subjected to chilling treatment, the DMRs peaks are 300-400 bp upstream of TSS (Zhang *et al.* 2016). In this study, the abundance peak of DMRs appeared in regions with a distance of 600-100 bp to TSS/TES. Moreover, DEGs showed clearly negative associations with DMRs, especially CG-DMRs near TSSs/TESs. These results support a causal relationship between cytosine methylation changes and transcription level changes. Unlike in vertebrates whose CpG island (CGI) features located at the transcription initiation site are markers for epigenetic regulation (Illingworth and Bird 2009), higher plants lack a unified CGI feature to predict epicontrol. Nevertheless, many studies demonstrate a negative influence of DNA methylation near the TSS/TES regions on gene activity, as demonstrated in bacterial infection (Dowen *et al.* 2012), nematode infection (Rambani *et al.* 2015), phosphate starvation (Yong-Villalobos *et al.* 2015), and application of the pesticide atrazine (Lu *et al.* 2016). DNA hypermethylation may prevent accessibility to regulatory factors and thereby regulate transcriptional activity.

We predicted possible epiregulation sites of DEGs based on the universal principle of negative association between gene expression and the DNA methylation level of regions adjacent to TSS/TES. Interestingly, a CHG-type DMR site was identified in the promoter region of an FLC-like MADS-box protein gene. Moreover, 10 TEs were identified in the 2-kb promoter region of this gene, although their transcription level change was not significant. In fact, we identified DETs based on mRNA-seq data, which means that the transcription level of many TEs cannot be detected. Nevertheless, the detected DETs show a strong association with DMRs (Table 1). Together, the presence of as many as 10 TEs in the promoter strongly supports the epigenetic regulation site of the FLC-like gene. Sex-specific histone modification of MADS-box genes was determined in melon (Latrasse *et al.* 2017), suggesting the coordinated modification of histone and DNA in the epiregulation of sex expression. It is likely that MADS-box genes are epiregulation targets in the plant ESD process. In contrast, epicontrol of ethylene biosynthetic genes might account for the temperature-dependent regulation of sex expression in cucumbers (Lai *et al.* 2017).

In addition to TSS/TES adjacent regions, we observed a negative association of gene expression and CG- and CHG-type DNA methylation change. CDS methylation is an ancient event and a general feature in organisms (Zemach *et al.* 2010). A similar phenomenon was observed in pesticide-treated rice (Lu *et al.* 2016). However, the cucumber genome has shown a positive correlation of CDS CG-methylation and gene transcription levels (Lai *et al.* 2017), and it has been documented that modestly transcribed genes are most likely to be methylated, whereas genes that show either extremely high or low transcription levels are least likely to be methylated (Zilberman *et al.* 2007). Moreover, CDS methylation is limited to the CG context in normal conditions (Cokus *et al.* 2008), but environmental stimuli apparently induce CHG- and CHH-type DNA methylation, as found in this study and others (Rambani *et al.* 2015). This observation seems to be a confusing contradiction. Unknown mechanisms may be responsible for the negative interaction of CDS methylation and gene expression. After all, a definite function of CDS methylation remains elusive, let alone environmentally induced methylation changes. It has been suggested that gene body methylation might regulate alternative splicing efficiency and prevent the aberrant transcription of long genes (Zilberman *et al.* 2007).

In summary, we characterized the transcriptome and methylome response to photoperiod condition change. The *in silico* analysis helped to predict the epiregulation site for photoperiod-dependent regulation of cucumber sex expression. In the near future, site-specific epimutagenesis can further provide steady experimental evidence for epigenetic regulation. Targeted DNA methylation approaches have been attempted by fusing DNA methylation catalytic enzymes to DNA-specific editing systems, such as zinc finger proteins (ZFPs) (Siddique *et al.* 2013) and transcriptional activator-like effector (TALE) (Bernstein *et al.* 2015). Recently, Morita *et al.* (2016) adopted the ScFv-GCN4 antibody-peptide pair called SunTag to improve the efficiency of CRISPR/Cas9-based site-specific DNA methylation modification up to 100% (Morita *et al.* 2016). Although all reports concern only animal cells, they also shed light on the resolution for plants.
